# Microalgae Characterization for Consolidated and New Application in Human Food, Animal Feed and Nutraceuticals

**DOI:** 10.3390/ijerph15112436

**Published:** 2018-11-01

**Authors:** Antonio Molino, Angela Iovine, Patrizia Casella, Sanjeet Mehariya, Simeone Chianese, Antonietta Cerbone, Juri Rimauro, Dino Musmarra

**Affiliations:** 1 ENEA, Italian National Agency for New Technologies, Energy and Sustainable Economic Development, Department of Sustainability—CR Portici. P. Enrico Fermi, 1, 80055 Portici (NA), Italy; antonio.molino@enea.it (A.M.); angela.iovine@unicampania.it (A.I.); patrizia.casella@enea.it (P.C.); sanjeet.mehariya@unicampania.it (S.M.); anto.cerbone@gmail.com (A.C.); juri.rimauro@enea.it (J.R.); 2 Department of Engineering, University of Campania “Luigi Vanvitelli”, Real Casa dell’Annunziata, Via Roma 29, 81031 Aversa (CE), Italy; simeone.chianese@unicampania.it

**Keywords:** market, bio-products, protein, lipids, carotenoids, *Arthospira platensis*, *Scenedesmus almeriensis*, *Haematococcus pluvialis*, *Chlorella vulgaris*, *Dunaliella salina*, *Nannochloropsis* sp.

## Abstract

The exploration of new food sources and natural products is the result of the increase in world population as well as the need for a healthier diet; in this context, microalgae are undoubtedly an interesting solution. With the intent to enhance their value in new commercial applications, this paper aims to characterize microalgae that have already been recognized as safe or authorized as additives for humans and animals (*Chlorella vulgaris*, *Arthrospira platensis*, *Haematococcus pluvialis*, *Dunaliella salina*) as well as those that have not yet been marketed (*Scenedesmus almeriensis* and *Nannocholoropsis* sp.). In this scope, the content of proteins, carbohydrates, lipids, total dietary fiber, humidity, ash, and carotenoids has been measured via standard methods. In addition, individual carotenoids (beta-carotene, astaxanthin, and lutein) as well as individual saturated, monounsaturated, and polyunsaturated fatty acids have been identified and quantified chromatographically. The results confirm the prerogative of some species to produce certain products such as carotenoids, polyunsaturated fatty acids, and proteins, but also show how their cellular content is rich and diverse. *H. pluvialis* green and red phases, and *Nannochloropsis* sp., in addition to producing astaxanthin and omega-3, contain about 25–33% *w*/*w* proteins on a dry basis. *D. salina* is rich in beta-carotene (3.45% *w*/*w* on a dry basis), *S. Almeriensis* is a source of lutein (0.30% *w*/*w* on a dry basis), and the *C. vulgaris* species is a protein-based microalgae (45% *w*/*w* on a dry basis). All, however, can also produce important fatty acids such as palmitic acid, γ-linolenic acid, and oleic acid. Considering their varied composition, these microalgae can find applications in multiple sectors. This is true for microalgae already on the market as well as for promising new sources of bioproducts such as *S. almeriensis* and *Nannochloropsis* sp.

## 1. Introduction

In recent years, there has been a growing interest in the application of microalgae as a new source of food and products for the human diet, due both to the increase in the population (projected to reach US$8.3 billion in 2030 [1]) and to the growing awareness and search for healthier foods. At the same time, the desire to reduce and/or replace synthetic compounds derived from fossil sources has led to the search for new natural resources from which to extract natural compounds to be used as food additives, as ingredients in drugs and nutraceuticals, or in cosmetics.

Microalgae are versatile cell factories that can produce different types of compounds such as proteins, carbohydrates, lipids, carotenoids, vitamins, and mineral salts according to growth conditions [2,3]. In recent years, their composition has been investigated by several authors who have compared the best known species, such as *Chlorella vulgaris*, *Arthrospira platensis*, *Nannochloropsis* sp., *Botryococcus braunii*, and *Phaeodactylum tricornutum* [4,5], other individual species such as *Dunaliella salina* [6], and *Haematococcus pluvialis* in the red phase [7] and green phase [8], and less studied species such as *Scenedesmus almeriensis* [9]. The characterization of microalgae cellular content allows us to learn not only the peculiarities of some species for producing larger quantities of some specific compounds such as long-chain fatty acids (in particular omega-3, eicosapanthenoic acid), carotenoids (such as beta-carotene and astaxanthin), and proteins, but also to identify their possible applications in different sectors.

The largest field of application for microalgae is in the food sector. Species such as *C. vulgaris* and *A. platensis* have already been recognized as generally recognized as safe (GRAS) by the US FDA (Food and Drug Administration) and by EFSA (European Food Safety Authority) [10,11,12,13]. These species are used in the food sector both as additives in food and beverages due to their protein content (between 42% and 58% *w*/*w* on a dry basis [14]), and for their content of phycobiliproteins (coloring substances with excellent antioxidant properties) which are mainly present in *A. platensis* [15]. Other compounds that are appreciated in the food field are carotenoids, in particular beta-carotene for its coloring and vitaminic properties. Being a precursor of provitamin A, beta-carotene is partly directly converted into retinol (vitamin A) once ingested in the intestinal tract [16]. *D. salina* is already a recognized source of beta-carotene from which carotene extract is produced with at least 80% beta-carotene, which is used as a coloring additive in food (E 160 IV). In addition, to coloring substances, lipids produced by microalgae are also used in the food industry. The most requested lipids are long-chain fatty acids, in particular omega-3, eicosapanthenoic acid (EPA), and docosapanthenoic (DHA), that, according to the World Health Organization, should be taken in portions of 200–500 mg to prevent cardiovascular disease (WHO 2003). The lipid content extracted from microalgae, in particular from the species *Ulkenia* sp. and *Schizochytrium* sp., is used to enrich omega-3 foods and drinks for lactating women and other adults.

Another interesting field for microalgae application is animal feed. The aquaculture sector is the main field for microalgae because they can be a source of high quality proteins, vitamins, carotenoids, and omega-3 [17]. Currently, the FDA has already authorized the use of *H. pluvialis* in the red phase as a coloring additive to feed shrimps and salmonids in the form of dry biomass or as astaxanthin extracts. Other microalgae that can be used in aquaculture to enrich the diet of shrimps and salmon with beta-carotene or with EPA and DHA are the species *D. salina* and *Nannochloropsis* sp. [18,19]. In addition, the proteins contained in microalgae can be an excellent food to be integrated into the diet of shrimps [20], salmon, and carp [21].

Microalgae are also used as food supplements in the nutraceutical field in the form of powders and tablets. *C. vulgaris* and *A. platensis* are the most widely known and used microalgae in the nutraceutical sector due to their content of vitamins, essential amino acids, and polyunsaturated fatty acids [22]. Other species used in the nutraceutical field as ingredients for food supplements are *D. salina* and *H. pluvialis* for their vitaminic and antioxidant properties [23].

In the cosmetics sector, the most interesting microalgae are *C. vulgaris* for the synthesis of collagen as an agent with anti-aging properties, and *D. salina* and *H. pluvialis* for their content of carotenoids as coloring agents and for UV protection. Meanwhile, microalgae, with an excellent production of lipids, can be used for the production of linoleic acid as an active ingredient for skin softness [24].

Microalgae have different applications. Their global market value was US$2.8 billion in 2011, with a production of 9000 tons/dry biomass. This market is expected to be worth US$3.32 billion by 2022, with a compounded average growth rate (CAGR) growth of 6.7% [25,26]. Food and feed together with food supplements dominate the global microalgae market, and dry microalgae biomass constitutes 75% of the nutraceutical market. Another promising market is that of carotenoids, which was estimated in 2015 to be worth US$1.21 billion. Of these, beta-carotene and astaxanthin are the most requested compounds in the human and animal food sector [27]. The omega-3 market was worth US$2.04 billion in 2016. Of this, 80% is due to the sale of food supplements and functional foods, and 10% is due to aquaculture [28]. One potential market is that of proteins, which at present has a value of US$0.596 billion, with a growth forecast of 7.03% CAGR by 2023 [29]. The global consumption of proteins could reach 944 million tons in 2054 due to rapid population growth. Eighteen percent of this could be sourced from proteins of plant origin to reduce the production of animal proteins from intensive farming [30]. Microalgae proteins have also hypocholesterolemic and hypoglyceridemic properties that prevent cardiovascular disease [31], and can be used as ingredients in foods against obesity [32].

Starting from the above-mentioned short literature overview some of these microalgae are already used in the commercial sector and their market value is based on the production of specific bio-products, such as astaxanthin for *Haematococcus pluvialis*, beta-carotene for *Dunaliella salina*, omega 3 for *Nannochloropsis* sp., and protein for *Chlorella vulgaris* and *Arthrospira platensis*. The objective of this paper is to provide a complete bio-products characterization in the above-mentioned microalgae and in some less-investigated species such as *Haematoccous pluvialis* green phase and the not yet-marketed *Scenedesmus almeriensis*.

## 2. Materials and Methods

The microalgal species were acquired as freeze-dried powder. *Chlorella vulgaris*, *Artrhospira platensis*, and *Haematococcus pluvialis* in red and green phase, and *Nannochloropsis* sp. were supplied by the company MICOPERI BLUE GROWTH^®^ (Ravenna, Italy). *Dunaliella salina* was provided by the company Algalimento (Santa Lucía de Tirajana, Gran Canaria) and *Scenedesmus almeriensis* was provided by the Company AlgaRes Srl (Rome, Italy). Each biomass was stored at −20 °C before characterization and analysis. Solvents such as ethanol, acetone, methanol, acetonitrile, isooctane, and chloroform were purchased by Sigma-Aldrich (Saint Louis, MO, USA) with a chromatographic grade; sulfuric and boric acids were purchased at ACS grade, and water was purchased at u-HPLC grade. For each investigation, three sample of microalgae biomass were treated (*n* = 3). Results are reported as average value ± standard deviation. Statistical analysis (One-Way ANOVA was performed using the statistical software SigmaStat 4.0 (Systat Software Inc., San Jose, CA, USA).

### 2.1. Moisture and Ash

The humidity and ash content were respectively determined by following the standard methods UNI EN ISO 712 [33] and UNI EN ISO 2171 [34], according to which a defined quantity of sample (5 g) was weighed up to constant weight before and after drying at 105 °C with incineration at 550 °C.

### 2.2. Proteins

The protein content was determined according to the Kjeldhal method, reported in the standard procedure UNI EN ISO 20483:2014 [35]. A defined quantity of lyophilized micro-algae powder (1 g) with a nitrogen content between 0.005 and 0.2 g was digested for 2 h at 420 °C using 10 g of Kjeldahl reagent (1.18348 EMD Millipores, Sigma-Aldrich Ltd., St. Louis, MO, USA) and 20 mL of sulphuric acid. After digestion, the sample was cooled and distilled with steam. In the collection flask, 50 mL of boric acid and 10 drops of color indicator were added for the final titration with sulfuric acid up to the color change point. The percentage of nitrogen in the dry weight obtained was multiplied by a factor of 6.25 for the determination of the protein content.

### 2.3. Carbohydrates

Carbohydrates were extracted from a defined amount of lyophilized microalgae (about 12 mg) by acid hydrolysis with H_2_SO_4_ (80% wt) for 20 h at 20 °C [36]. The total content of carbohydrates was analyzed using an u-HPLC coupled with an ELSD detector, following the chromatographic conditions reported in the standard method UNI EN ISO 15086 [37].

### 2.4. Lipids

The lipid content was extracted from about 120 mg of each lyophilized microalgae using a mixture of chloroform:methanol:distilled water with a ratio of 1:2:0.75 (3.75 mL) in continuous stirring for 1 h using the Bligh and Dyer method as modified by Tang et al. [38]. After extraction, 1 mL of chloroform and 0.5 mL of double-distilled water were added. The sample was centrifuged at 14,000× rpm for 10 min and the upper phase was taken and stored in a tube. The extraction was repeated on the initial biomass until the color was lost and the extract obtained was combined with the previous extract. The total content of lutein was gravimetrically quantified, after the removal of the solvent using a Zymark TurboVap evaporator (Zymark, Hopkinton, MA, USA). The extracts obtained after the Bligh and Dyer extraction were transesterified according to the indications given in the standard method UNI ISO 12966-2 [39]. NaOH solution in methanol (0.5 M, 6 mL) and a spatula of boiling chips were added to a known quantity of extracted oil (250–500 mg). The sample was transferred to a 50 mL one-mark volumetric Erlenmeyer flask connected to a reflux condenser to boil the sample for about 10 min. At the end of boiling, the apparatus was removed from the heat source and 6 mL of hexane was added from the top of the condenser and then 7 mL of the BF3 catalyst in methanol (14%) (B1252 Aldrich, Sigma-Aldrich Ltd., St. Louis, MO, USA). The sample was boiled again for 30 min and 5 mL of isooctane was added at the end of the reaction. A 20-mL sample of a saturated NaCl solution was added and swirled, and a second aliquot of saturated NaCl solution was added until the neck of the flask. The upper layer (2–4 mL) was taken and transferred to a GC glass vial. The chromatographic analysis was carried out using a 7820A GC-FID equipped with an HP-88 100 mt × 0.25 mm × 0.2 µm column. According to the chromatographic conditions reported in the standard method UNI ISO 12966-4 [40], the temperature of the injector and detector were programmed at 250 °C and that of the oven from 150 °C to 240 °C with a ramp of 4 °C min. Nitrogen (purity > 99.999%) was used as a gas carrier with a spatial velocity of 30 cm/s. An internal analytical standard, the heneicosanoic acid (C:21) purchased form SIGMA-Aldrich (H5149) (Sigma-Aldrich Ltd., St. Louis, MO, USA) was used to quantify fatty acid methyl esters and a mixture of 37 fatty acid ethyl esters (C4–C24) (Supelco FAME 37, CRM47885, Sigma-Aldrich Ltd., St. Louis, MO, USA) was used for the qualitative analysis.

### 2.5. Total Dietary Fiber

Total fiber content was determined both enzymatically and gravimetrically by the standard method AOAC 985.29 [41]. The lyophilized microalgae sample was previously homogenized by a mill for 1 min at 200× rpm. Each portion was greased three times with 25 mL of light petroleum per sample rate when the lipid content was greater than 10%. After sample preparation, about 1 g of sample was transferred to a 400-mL beaker, where 50 mL of phosphate buffer was added at pH 6.0. The sample was initially digested by adding 0.1 mL of Termamyl (heat-stable alpha-amylase, A4862 Sigma, Sigma-Aldrich Ltd., St. Louis, MO, USA) for 15 min in boiling water at a temperature between 95 and 100 °C; then the pH was changed to 7.5 before adding 5 mg of protease (P3910 Sigma). The sample was incubated for 30 min at 60 °C under continuous shaking. Finally, 0.3 mL of aminoglucosidase (A9913 Sigma) was added to the sample after the pH was in the range of 4.0–4.6. The sample was incubated again at 60 °C for 30 min under continuous shaking. At the end of the incubation, 280 mL of 95% ethyl alcohol was added and previously heated to 60 °C, and the sample was left at room temperature and precipitated for 1 h. The sample was filtered on a bed of Celite contained in a fritted crucible, whose weight, funnel and Celite were previously noted. The residue deposited on the Celite bed was washed sequentially with three 20-mL aliquots of 78% ethyl alcohol, two 10-mL aliquots of 95% ethyl alcohol, and three 20-mL aliquots of acetone, keeping the filtration apparatus turned on. After washing, the funnel was dried at 105 °C overnight and weighed after cooling in a dryer to determine the dry weight of the digested sample. The funnel was then incinerated at 525 °C for 5 h to determine the ash.

### 2.6. Carotenoids

Carotenoids were extracted from *D. salina*, *H. pluvialis* in the red phase, and *S. almeriensis* following the analytical procedure described by Li et al. [42]. A defined amount of dry biomass (about 20 mg) was manually grinded until completely discolored and the astaxanthin content was extracted using 10 mL DMSO. The sample was centrifuged to separate the remaining biomass from the liquid extract, which was then combined with the previous extract.

The total content of carotenoids was quantified using an Agilent 1290 Infinity II uHPLC equipped with a diode array Detector (DAD) for detection in both the 400–700 nm wavelength range and at certain wavelengths such as 444 nm, 450 nm, 478 nm for the identification and quantification of individual species such as lutein, beta-carotene, and astaxanthin [43]. Chromatographic analysis was performed using an Agilent Zorbax Eclipse plus C18 column 1.8 μm column, with an isocratic mobile phase with acetonitrile and methanol (85:15 *v*/*v*) at a flow of 0.8 mL/min and a temperature of 30 °C for the identification of beta-carotene, as indicated in the standard method UNI EN 12823-2 [44], while for the analysis of astaxanthin and lutein a methanol:water (95:5) isocratic phase was used at a flow rate of 0.4 mL/min and a temperature of 28 °C [45]. For the quantification of each species, astaxanthin (SML0982 SIGMA), beta-carotene (22040 Sigma), and lutein (07168 Sigma-Aldrich) analytical grade standards were used, which were dissolved in chloroform with 0.1% butylated hydroxytoluene (BHT) as the antioxidant.

## 3. Results and Discussion

### 3.1. Microalgae Composition

**Figure 1:**
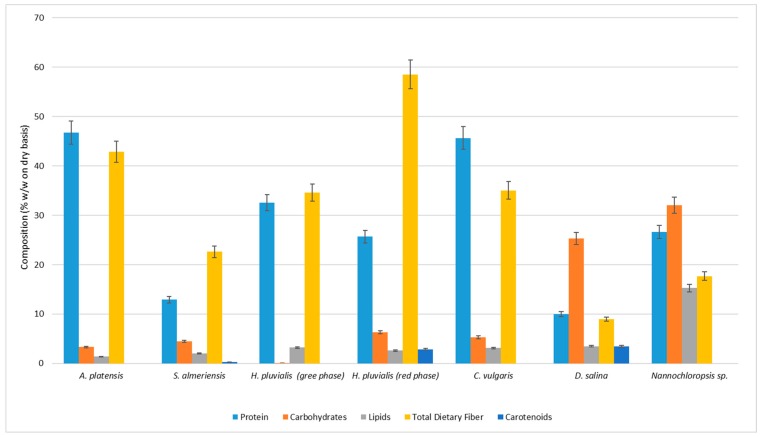
Protein, carbohydrates, lipids, total dietary fiber and carotenoids compositions (*w*/*w* on a dry basis ± SD (*n* = 3)) of *Arthrospira platensis platensis*, *Scenedesmus almeriensis*, *Haematococcus pluvialis* (green phase), *Haematococcus pluvialis* (red phase), *Chlorella vulgaris*, *Dunaliella salina*, *Nannochloropsis* sp.

Microalgae were characterized both in terms of moisture and ash, reported in Table 1 and in terms of proteins, carbohydrates, lipids, total dietary fiber, and carotenoids, reported in Table 2 and Figure 1.

The highest moisture content, expressed as a percentage of the wet weight, unlike the other parameters, was observed for the three species *Scenedesmus almeriensis*, *Arthrospira platensis*, and *Dunaliella salina* and equal to 8.89 ± 0.32%, 6.45 ± 0.15% and 6.63 ± 0.25%, respectively (Table 1). The microalgae *Haematococcus pluvialis* had a higher moisture content in the green phase (5.03 ± 0.12%) than in the red phase (2.79 ± 0.23%), while the species with the lowest water contents were *Nannochloropsis* sp. and *Chlorella vulgaris* with 1.90 ± 0.05% and 1.92 ± 0.09%, respectively (Table 1).

The highest ash content was found in the microalgae *S. almeriensis* and *D. salina,* with a percentage of 57.61 ± 2.20% *w*/*w* on a dry basis and 48.74 ± 2.50% *w*/*w* on a dry basis, respectively (Table 1). Such a high percentage of ash can be related to the presence of salts on the surface of the dry biomass [5] since *D. salina* is a halotolerant species that lives in a highly saline environment, while the high amount of ash for *S. almeriensis* is related to the high content of alkali and alkali-earth metals [46]. The microalgae *H. pluvialis* in green phase has an ash content of 29.49 ± 0.22% *w*/*w* on a dry basis, while in the remaining microalgae have lower values, equal to 8.31 ± 0.42% *w*/*w* on a dry basis in *Nannochloropsis* sp., 10.88 ± 0.51% *w*/*w* on a dry basis in *C. vulgaris*, 4.02 ± 0.22% *w*/*w* on dry basis in *H. pluvialis* in the red phase, and 5.71 ± 0.32% *w*/*w* on a dry basis in *A. platensis* (Table 1).

The highest protein content was found in the microalgae *A. platensis* and *C. vulgaris* with a percentage of 46.76 ± 0.95% *w*/*w* on a dry basis and 45.46 ± 1.20% *w*/*w* on a dry basis, respectively (Figure 1).

As shown in Figure 1, the microalgae *H. pluvialis* had a higher content of proteins in the green phase (32.59 ± 1.20% *w*/*w* on a dry basis) compared to the red phase (25.69 ± 1.27% *w*/*w* on a dry basis) which fell within the range reported in other works [7,8]. The percentage of proteins observed in *Nannochloropsis* sp. (26.67 ± 1.10% *w*/*w* on a dry basis) was slightly higher than that found in *H. pluvialis* in red phase (Figure 1) and *D. salina* and *S. almeriensis* were the species with the lowest percentage of proteins.

The highest amount of carbohydrates was observed in *Nannochloropsis* sp. and *D. salina* that contained 32.05 ± 0.70% *w*/*w* on a dry basis and 25.31 ± 1.55% *w*/*w* on a dry basis, respectively (Figure 1). In other microalgae species, the percentage of carbohydrates is much lower, since *C. vulgaris* contained up of 5.30 ± 0.50% of the dry weight and *A. platensis* has a slightly lower content (3.32 ± 0.05%) (Figure 1).

In *H. pluvialis*, there was a difference carbohydrates content between the green and red phases (Figure 1). In this case, unlike for proteins, the percentage of carbohydrates is lower in the green phase than in the red phase. The ability of the microalgae *Nannochloropsis* sp. to produce lipids is also evident in our work since the highest percentage of lipids equal to 15.30 ± 0.24% *w*/*w* on a dry basis has been observed in this species (Figure 1). With regard to the other species, no substantial differences were observed; however, the descending order is as it follows: *D. salina* > *H. pluvialis* > *C. vulgaris* > *H. pluvialis* red phase > and *A. platensis* (Figure 1).

The highest total dietary fiber content was found in the microalgae *H. pluvialis* in the red phase with a percentage of 58.52 ± 2.56% *w*/*w* on a dry basis (Figure 1). The high percentage of fiber is due to the structural characteristics of the cell wall of the cyst, which is mainly made up of sporopollenin, a complex and very resistant polysaccharide [47]. *A. platensis* and *C. vulgaris* have a fiber content slightly lower than *H. pluvialis* in the red phase of 42.82 ± 1.20% *w*/*w* on a dry basis and 35.04 ± 1.60% *w*/*w* on a dry basis, respectively (Figure 1). *D. salina* shows the lowest percentage, equal to 8.97 ± 0.50% (Figure 1).

### 3.2. Carotenoids

The total carotenoid content has been quantified in the specie *D. salina*, *H. pluvialis* in the red phase, and *S. almeriensis* since they are the microalgae with the highest ability to produce these compounds.

**Figure 2:**
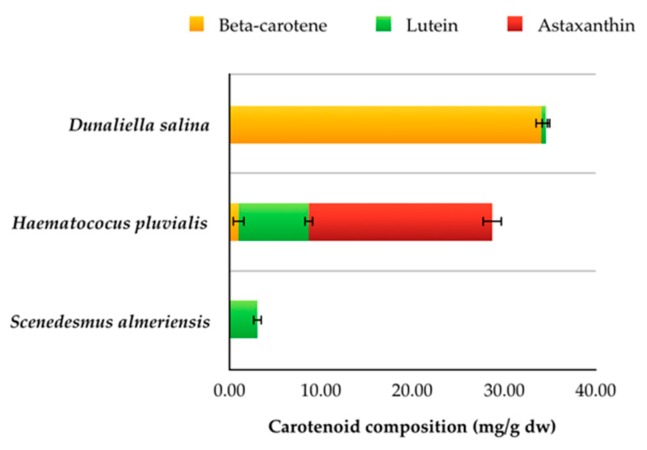
Beta-carotene, lutein and astaxanthin quantified in the three species *Dunaliella salina*, *Haematococcus pluvialis* (red phase), and *Scenedesmus Almeriensis* (the values are expressed in mg/g on dry weight ± SD (*n* = 3)).

As can be seen in Table 2, *D. salina* contains the highest percentage of total carotenoids of 3.46 ± 0.15% *w*/*w* on a dry basis, whose value is very close to the percentage observed in Garcìa-Gonzalez et al. [48] such as 4% *w*/*w* on a dry basis, and higher than the range of values 0.8–2.78% *w*/*w* on dry basis reported in other works [49,50]. In *H. pluvialis* (red phase) total carotenoids constitute 2.87 ± 0.15% *w*/*w* on a dry basis, as reported in other works [7,51]. *S. almeriensis* has a lower content, with 0.30 ± 0.05% *w*/*w* on a dry basis of total carotenoids with a capacity to accumulate larger amounts of them up to 1.38% on a dry basis [9]. In addition to the amount of total carotenoids, the composition of individual carotenoids such as beta-carotene, astaxanthin, and lutein, expressed in mg/g on a dry basis, was also investigated (Figure 2).

Since *D. salina* is the largest producer of beta-carotene, this constitutes 98.58% of the total carotenoids, with a quantity equal to 34.10 ± 0.7 mg/g on a dry basis and a small percentage consists of lutein equal to 0.49 ± 0.05 mg/g on a dry basis. The production of beta-carotene depends very much on the growth conditions. *D. salina* is able to accumulate beta-carotene even up to 70 mg/g on a dry basis under high light intensity (1000 µmol m^−2^ s^−1^) and nitrogen depletion, while in conditions of low light intensity (100 µmol m^−2^ s^−1^) and high salinity (4 M NaCl) it can contain about 35 mg/g on a dry basis [52].

Astaxanthin is the main carotenoid produced by the *H. pluvialis* red phase, constituting 81.2% of total carotenoids [7]. This compound accounted for 69.72% of total carotenoid with a value of 20.01 ± 1.05 mg/g on a dry basis. In addition, *H. pluvialis* the red phase also contains 7.70 ± 0.07 mg/g on a dry basis of lutein and 0.99 ± 0.6 mg/g on a dry basis of beta-carotene. Lutein and beta-carotene were often found in the literature in lower percentages, such as 1% and 0.5% of total carotenoids, respectively [7]. The amount of astaxanthin produced in *H. pluvialis* can reach up to 43 mg/g on a dry basis in heterotrophic cultivation with carbon sources in the form of acetate to autotrophic conditions [53].

The microalgae *S. almeriensis* is confirmed as the largest producer of lutein with a content of 3.04 ± 0.35 mg/g on a dry basis with the ability to produce even greater quantities equal to 4.5 mg/g on a dry basis [54] and 5.5 mg/g on a dry basis [55].

### 3.3. Fatty Acid Composition

**Figure 3:**
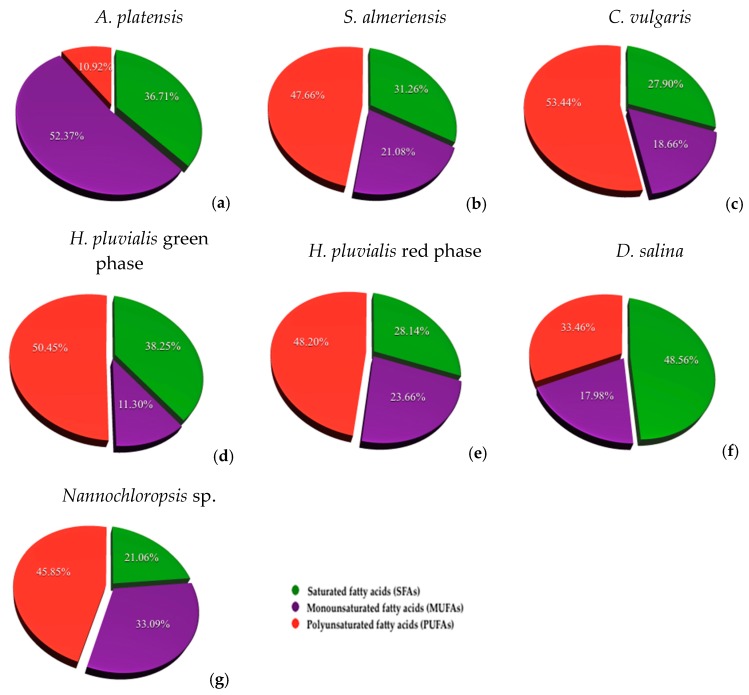
Fatty acids compositionexpressed as saturated fatty acids (SFAs), monounsaturated fatty acids (MUFAs) and polyunsaturated fatty acids (PUFAs) (the pie charts are presented from left to right, from the top row to the bottom row on the basis of the increasing order of the total amount of fatty acids): (**a**) *Arthrospira platensis*; (**b**) *Scenedesmus almeriensis*; (**c**) *Chlorella vulgaris*; (**d**) *Haematococcus pluvialis* (gree phase); (**e**) *Haematococcus pluvialis* (red phase); (**f**) *Dunaliella salina*; (**g**) *Nannocholoropsis* sp. The values are reported in percentages. A standard deviation of less than 5% was found (*n* = 3).

Fatty acid composition was initially classified into saturated (saturated fatty acids—SFAs), monounsaturated (monounsaturated fatty acids—MUFAs) and polyunsaturated (polyunsaturated fatty acids—PUFAs) classes whose percentages of the total are shown in Figure 3 while the identification and quantification of individual compounds is reported in Table 3.

The lowest quantity of fatty acids was observed in the microalgae *A. platensis*, equal to 1056.06 ± 4.17 mg/100 g of dry biomass. Among total fatty acids, monounsaturated (MUFAs) constituted the 52.37% and palmitic acid is the most abundant as observed in other works [5,56]. Apart from palmitic acid, linoleic and γ-linolenic acid can also be the most widely produced fatty acids in *A. platensis* [5,57,58]. In *S. almeriensis* we found a higher percentage of PUFAs (47.66%) and a slight increase in the quantity of fatty acids equal to 1116.78 ± 4.67 mg/100 g of dry biomass. γ-Linolenic acid is the most abundant, equal to 370.84 ± 0.84 mg/100 g of dry biomass as reported by Sanchez et al. [59]. Fatty acids increased significantly in *C. vulgaris* with 2370.13 ± 4.39 mg/100 g of dry biomass, with a higher percentage of PUFAs (53.44%). *H. pluvialis* in the red phase showed an higher amount of fatty acids (2297.88 ± 5.39 mg/100 g of dry biomass) with respect to *H. pluvialis* in the green phase (1931.79 ± 5.06 mg/100 g of dry biomass), and PUFAs are, in both cases, the most abundant classes, as already observed in other works [8,51]. In *D. salina*, total fatty acids represented around 3156.21 ± 2.27 mg/100 g of the dry biomass with a prevalence of palmitic acid, linoleic acid and γ-linolenic acid that was also observed in other works under the absence of nitrogen sources [60] or under the effect of high light intensities [52]. The highest amount of fatty acids was found in *Nannochloropsis* sp. with 10,999.75 ± 7.91 mg/100 g of dry biomass, with a percentage of PUFAs equal to 45.85%.

In the investigated species, the most widely distributed and abundant FAMEs are palmitic acid among saturated classes, oleic and myristoleic acid among the monounsaturated class, and linoleic acid and γ-linolenic acid among the polyunsaturated class. Palmitic acid is very abundant in *Dunaliella salina* (965.00 ± 1.15 mg/100 g of dry biomass) followed by *Chlorella vulgaris* (598.75 ± 2.20 mg/100 g of dry biomass), and *Haematococcus pluvialis* green and red phase (*p* < 0.05). Oleic acid was quantified in all species except to *Haematococcus pluvialis* green phase and *Nannochloropsis* sp. Its content is very variable between the microalgae, with a statistically significant difference (*p* < 0.05). In *D. salina* and *H. pluvialis* red phase, the highest oleic acid content was observed equal to 567.56 ± 1.29 mg/100 g of dry biomass and 486.17 ± 0.23 mg/100 g of dry biomass, respectively (*p* < 0.05). The higher oleic acid content in *D. salina* with respect to other species seems to be related to the carotengenesis process [61]. Myristoleic acid is predominantly abundant in *Nannohloropsis* sp. (483.93 ± 0.86 mg/100 g of dry biomass) compared to the species *Haematococcus pluvialis* green phase, *Chlorella vulgaris Arthrospira platensis*, and *Scenedesmus almeriensis* (*p* < 0.05). The highest linoleic acid content was found in *Haematococcus pluvialis* red and green phase, with a statistically significant difference compared to other species (*p* < 0.05). In contrast, the amount of γ-linolenic acid is significantly higher in *Haematococcus pluvialis* green phase (723.07 ± 0.21 mg/100 g of dry biomass) than red phase (205.85 ± 0.45 mg/100 g of dry biomass) (*p* < 0.05). A great content of γ-linolenic acid resulted also in *C. vulgaris* and *D. salina*.

Ecosapentaenoic acid (EPA, C20:5 (ω3)) was found only in *Nannochloropsis* sp., with a content equal to 3650.82 ± 1.97 mg/100 g of dry biomass. *Nannochloropsis* sp. contained also others fatty acids not detected in other species as heptadecanoic acid (C17:0) and palmitoleic acid (C16:1).

Van Wagenen et al. [62] also observed that palmitic acid, palmitoleic acid, and EPA were the most abundant compounds and that the concentration of EPA decreased under the effect of higher light intensity.

### 3.4. Comparison with Literature About Microalgae Composition

The microalgae characterization obtained from the investigated species was compared with data in the literature, as reported in Table 4.

The species that are richer in terms of protein content are *A. platensis* and *C. vulgaris.* This property is well recognized since they were among the first species to be industrially produced for food purposes. *A. platensis* is able to produce even higher percentages of protein between 50% *w*/*w* on a dry basis and 55% *w*/*w* on a dry basis [4,63] and, according to some authors even up to 63% *w*/*w* on a dry basis [64,65]. Results compared to our work have been observed by Matos et al. [5] with a percentage of protein around 42.08 ± 0.10% *w*/*w* on a dry basis. *C. vulgaris* can produce up to 60.38% *w*/*w* protein on a dry basis in growth conditions rich in nitrogen, while in the absence of nitrogen the presence of proteins can decrease up to 20% *w*/*w* on a dry basis [67]. The protein content in this species can also be influenced by the percentage of CO_2_ feed. The increase of CO_2_ from 1% to 10% (*v*/*v*) leads to an increase in protein from 25.50% *w*/*w* on a dry basis to 48.19% *w*/*w* on a dry basis [68]. Literature data about proteins in *Haematococcus pluvialis* in the red and green phase are not abundant. The comparison with the obtained results showed that protein in *H. pluvialis* red phase can also be lower in the range of 10.2–17% *w*/*w* on a dry basis [7,51]. The protein content observed in literature about *Nannochloropsis* sp. are discordant, with an average value of 28.8 ± 0.63 in Rebolloso-Fuentes et al. [72] and a range of 41.6–42.1% [5]. *D. salina* and *S. almeriensis* can also produce higher percentages up to 55% *w*/*w* on a dry basis depending on nitrate concentrations in the growth medium [49]. The composition of *S. almeriensis* has so been little studied in terms of protein, and the content found in our work is lower than the values reported in literature (49.4% *w*/*w* on a dry basis) because of the different growth conditions [59]. *Chlorella vulgaris* is a microalgae with a carbohydrates content of 59.71% *w*/*w* on a dry basis that is not comparable with our results [68,69].

According to our work, *D. salina* and *Nannochloropsis* sp. produced the higher content of carbohydrates. Carbohydrates may be lower in some species such as *Nannochloropsis oculata* and *Nannochloropsis gaditana*, with percentages of 16.7% *w*/*w* on a dry basis and 18.6% *w*/*w* on a dry basis [5]. Their content can be influenced by the retention time of 4.8 and 14.5 days, respectively, within a 29 L cylindrical photobioreactor [71]. The percentage of carbohydrates seems to be variable also in *D. salina* under nitrogen nutrient deprivation after 20 h of cultivation, in which the microalgae produces 25% *w*/*w* on a dry basis respect to a content of 40% *w*/*w* on a dry basis under nitrate supply [49]. In *A. platensis*, carbohydrates content can be very variable with low values as 7.7% *w*/*w* on a dry basis [65] until to 22.2% *w*/*w* on a dry basis [4]. The higher content of carbohydrates in *H. pluvialis* red phase with respect to the green phase is due to the reserve function of this compounds in starvation conditions [7]. The carbohydrate content in *S. almeriensis* has been less investigated but this microalgae could produce up to 24.6% *w*/*w* on a dry basis [9].

Among the studied species, *Nannochloropsis* sp. can accumulate more than 40% of lipids [49] and, its content is strongly dependent on growth conditions. In particular, high light intensity can induce a consistent increase of the production of total lipid and triacylglycerols (TAGs) [73,74]. The photoperiod can be also among the factors that affect lipid production, which decreases by up to 25.6% *w*/*w* on a dry basis when the photoperiod is 24 h and increases by up to 30% when the photoperiod is 18 h light: 6 h dark cycle [72]. In addition, other factors that can affect lipid production in *Nannochloropsis* sp. are nitrate deprivation and salinity [75]. Temperature and harvesting time can also influence total lipid content in *Nannochloropsis salina* and *oculata* [76].

*D. salina* besides being a potential resource of beta-carotene, can also accumulate a good percentage of lipids up to 17% dry weight in particular growth conditions such as high light intensity and temperatures of 37 °C [70]. The percentages of lipids found in other species are lower than those reported in the literature but may vary in relation to different growth conditions, from 12% *w*/*w* on a dry basis to 26% *w*/*w* on a dry basis in *C. vulgaris*, from 4% *w*/*w* on a dry basis to 9% *w*/*w* on a dry basis in *A. platensis*, and other species belonging to the genus *Scenedesmus* may constitute even only 1.58% *w*/*w* on a dry basis [66].

Total dietary fiber in fact consists of complex polysaccharides that are more or less digestible by humans, such as starch or lignin. The higher fiber content in *Haematococcus pluvialis* red phase is due to the polysaccharidic nature of the cell wall in the red cyst. The percentage observed in *A. platensis* is in line with the value reported by Gershwin and Belay [64] but can also reach lower values equal to 8.5% *w*/*w* on a dry basis [5]. The fiber content in *C. vulgaris* is variable at the same time, with values equal to 5.6% *w*/*w* on a dry basis [5], and between 16.37% and 25.95% *w*/*w* on a dry basis [68]. Less comparative data were found for the other species because total dietary fiber is a typical analysis for food and feed applications.

In addition, microalgae composition was compared with cereals, the most used food for humans such as wheat, barley, rye, brown rice, sorghum, oats, maize. In Table 5, cereal composition is reported in term of moisture, ash, proteins, carbohydrates, lipids, total dietary fiber, and carotene.

The studied microalgae were found in the form of dry powder with a lower moisture content respect to cereals. The ash content is comparable to cereals in some microalgae such as *A. platensis*, *H. pluvialis* red phase and *Nannochloropsis* sp. is. It is sufficient to compare the protein content of *A. platensis* (46.76 ± 0.95% *w*/*w* on a dry basis), *Chlorella vulgaris* (45.64 ± 1.20) and other species such as *H. pluvialis* red and green phase, and *Nannochloropis* sp. to observe how their protein content is higher respect to cereals with a statically significant difference (*p* < 0.05), thus they could become potential sources of protein. Only protein content in microalgae *Nannochloropsis* sp. resulted not statistically different to cereals proteins (*p* > 0.05). The most comparable carbohydrate content with respect to cereals has been found in the microalgae *D. salina* and *Nannochloropsis* sp. (*p* < 0.05). Given the polysaccharide composition of cereals is rich in starch, lignin and hemicellulose, their carbohydrate content is expected to be higher. The cereals with the highest lipid content are oats and maize, with 4.9% and 5.9% *w*/*w,* respectively, based on dry weight, while in the case of microalgae, as we discussed earlier, the lipid content can reach 15.3 ± 0.24% *w*/*w* on a dry basis in species such as *Nannochloropsis* sp. [77]. Furthermore, species such as *D. salina*, *C. vulgaris*, and *H. pluvialis* green phase also showed an higher lipid content than other cereals, such as barley and sorghum. The total dietary fiber content of cereals is lower in brown rice and barley at about 4–5% *w*/*w* based on dry weight, and can reach up to 10.5–14.4% *w*/*w* based on dry weight in wheat and other cereals [78]. Among the species studied, *D. salina* and *Nannochloropsis* sp. had a total dietary fiber content comparable to the cereals (*p* < 0.05). If we consider wheat bran, a typical by-product of wheat milling, the amount of fiber can be around 52.2 ± 0.07% and 59.3 ± 1.9% *w*/*w* based on dry weight [79] and up to 78.4% *w*/*w* on a dry basis [80]. This by-product has found an application in livestock feed. Hence, the microalgae that contained a comparable fiber content, that could be less digestible for human can be considered for the application in livestock feeding. By contrast, the carotenoid content in cereals was significantly lower than in microalgae, except for maize (at 0.37% *w*/*w* based on dry weight). In addition, linoleic, oleic, and palmitic acid are found in oils derived from certain cereals such as corn, wheat, rye, and rice. Linoleic acid accounts for between 35% and 48% of total fatty acids, while oleic acid for 31% and 44% [77]. Among the microalgae investigated, *H. pluvialis* red phase contains the largest amount of linoleic acid which constitutes 31.09% of total fatty acids, while *D. salina* contains 30.57% of palmitic acid. These percentages are in line with those reported in the oils of some cereals.

### 3.5. Microalgae Market and New Challenges

Microalgae mainly dominate the market for food additives and food supplements. Food additives can be used either in the form of dry biomass powder or as extracts from bioproducts such as carotenoids and oils containing omega-3. Food supplements, on the other hand, are mainly produced as powders and in formulations such as tablets and oils. Production and marketing of microalgae-based products for food and feed are mainly regulated into the European Community by the Food Safety Regulation (EC 178/2002) and the Novel Food Regulation (EC 258/97); micro-algae-based food and feed products, once they are sold on the U.S. consumer market, are regulated by the Federal Food, Drug and Cosmetic Act (FD&C), introduced in 1938, and by the Dietary Supplement Health and Education Act, introduced in 1994 [25]. The main microalgae products currently authorized in Europe and by the U.S. FDA in the food and nutraceutical sectors and their costs are listed in Table 6. Microalgae products cost are very variable since they are affected by the composition of the microalgae in term of quantity of commercialized by-products (i.e., the percentage of astaxanthin, of EPA/DHA content). Another important factor that the influences the cost is the type of cultivations (open or closed systems).

*A. platensis* is one of the first species authorized in the food fields, whose extract containing phycocyanins is used as an additive and food supplement. Phycocyanins belong to the group of phycobiliproteins, a protein-chromophore complex with antioxidant properties that reduces peroxyl radicals, that has anti-inflammatory properties, and that reduces total cholesterol [87]. *A. platensis* had also an excellent protein content (>45% *w*/*w* on a dry basis). *C. vulgaris* contains not only a great protein content but also a rich quantity of fatty acids, such as linoleic, γ-linolenic, and palmitic acids. For these characteristics both species are highly appreciated as food supplements in the form of powder and tablets, as well as noodles, with commercial production of 2000 tons per year for *C. vulgaris* and 3000 tons per year for *A. platensis* [88]. Their protein content has also been recognized by the FAO for the presence of essential amino acids such as lysine, leucine, tryptophan in larger amounts than some typical protein foods such as legumes and meat [89].

Other species, such as *Tetraselmis chuii* and *Odontella aurita*, have been authorized as flavor enhancers and food additives in the form of dry biomass, while *Spongiococcum* and *H. pluvialis* are used respectively as feed additives for chickens and salmonids. *H. pluvialis* in the red phase has found its niche application in the animal feed market, and in particular in aquaculture as an additive for salmon both in the form of dry meal, i.e., as dry and comminuted material, and in the form of oleoresin rich in astaxanthin and extracted using the CO_2_-SFE technique. Considering that the astaxanthin content in the meal form must not be less than 1.5%, this is in line with the percentages found in the literature [7]. The market for astaxanthin has a value that was estimated in 2016 at US$555.4 million, unfortunately dominated by synthetic forms. In order to promote the natural forms of astaxanthin produced by the microalgae *H. pluvialis*, in recent years there has been implementation of green and safe extraction techniques with the use of generally recognized as safe (GRAS) solvents such as ethanol and acetone [90,91] or extraction with CO_2_-SFE [43,92]. *H. pluvialis* green phase and red phase could also be used in the feed additives market as a source of polyunsaturated fatty acids that make up most of their fat content, as has been observed in our and other works [8,51]. Furthermore, Ju et al. [20] proposed the use of the microalgae by-product obtained after the extraction of astaxanthin for nutraceutical purposes by means of CO_2_-SFE, i.e., a defatted biomass as an ingredient for shrimp feed given its content of proteins (40.3% *w*/*w* on a dry basis), lipids (0.9% *w*/*w* on a dry basis), and fiber (9.6% *w*/*w* on a dry basis). The authors have shown that the use of this biomass as a feed additive stimulates shrimp growth.

Since *D. salina* can have a carotenoid content of up to 4% *w*/*w* on a dry basis (of which beta-carotene accounts for 98.6% of total carotenoids), it is used as a source of a food additive (E-160 IV) consisting of an essential oil with a content greater than 20% beta-carotene and which may also contain other carotenoids such as lutein. With regard to the carotenoid market, lutein is another of the compounds already authorized and used as a food additive (E 161 b), but which is currently extracted with solvent from edible fruits and plants such as alfalfa and the *Tagetes erecta* plant. The flowers of the plant *Tagetes erecta* are currently the largest producers of lutein, which can constitute between 0.08% and 2.8% *w*/*w* on a dry basis, but which require a laborious process of extraction and have a production related to the seasonality of the plant [93]. A recent challenge could be to propose the inclusion of *S. almeriensis* in the lutein market. This market was worth US$135 million dollars in 2015, with a growing trend given the demand in the nutraceutical sector as a supplement for eye health thanks to its properties in reducing the risk of a serious disease such as age-related macular degeneration (AMD) caused by the decrease in pigments such as lutein, zeaxanthin present in the eye macula [94]. *S. almeriensis* can certainly be considered a new source of lutein, since it can accumulate between 0.3% and 0.54% *w*/*w* on a dry basis of lutein and it is grown indoors and outdoors without any difficulty related to seasonality as *Tagetes erecta* plant [9,55,59].

Another promising microalgae product is omega-3-rich oils such as EPA and DHA. Those currently authorized at European level in the food sector are produced by *Ulkenia* sp. and *Schizochytrium* sp. with an EPA and DHA content of more than 32% and 22.5%, respectively. The oil of the microalgae *Schizochytrium* sp. is mainly used for nutraceutical purposes while that produced by *Ulkenia* sp. in addition to being recognized as GRAS in the food industry, is also used in the cosmetics industry in skin care products. *Nannochloropsis* sp. could be the next type of microalgae to enter the omega-3 market, regarding the use of EPA in food and nutraceutical. This microalgae species has a usually higher lipid content than other microalgae (37–60% *w*/*w* on a dry basis) [75,95] and, as observed, EPA has a percentage (33.19% *w*/*w* on a dry basis) in line with the content required in the oils of *Ulkenia* sp. and *Schizochytrium* sp., according to Regulation (EU) 2017/2470 on novel foods. In addition, *Nannochloropsis* sp. could already be tested in the form of freeze-dried biomass as an ingredient for the preparation of biscuits and pasta enriched with omega 3, using about 2–3% biomass [96] or in some cases replacing 30% of durum wheat [97]. *Nannochloropsis* sp. is also a promising source of food for the aquaculture sector. Sørensen et al. [19] showed that 10% of the defatted biomass of *Nannochloropsis* sp. can be fed into the diet of Atlantic salmon without any negative effect on salmonid health or performance. The biomass composition consisted mainly of protein for 43.3% *w*/*w* on a dry basis, carbohydrates 28.8% *w*/*w* on a dry basis, and lipids 2.5% *w*/*w* on a dry basis, which were added to fish meal and fish oil, and wheat. Gbadamosi and Lupatsch [98] observed that replacing fishmeal and soya in the diet of the species *Oreochromis niloticus* (Nile tilapia) with *Nannochloropsis* sp. is possible to obtain the same growth performance, and a product with better nutritional values in terms of the composition of fatty acids. Vizcaíno et al. [99] have instead tested the species *S. almeriensis* as an ingredient in the diet of juvenile sea bream (*Sparus aurata*) demonstrating the possibility of using this species for 12% and 20% in feed without any negative effect on fish health. In addition, the species *C. vulgaris* and *A. platensis* have been tested as feed for poultry, ruminants, and pigs without any negative effect on animal health and in some cases improvements in meat quality have also been observed [100].

A new challenge is to use microalgae proteins as potential food sources and as hydrolyzed protein concentrates to be added to food and beverages. The protein content should be extracted by mechanical and/or physical and/or chemical pretreatment processes, such as high pressure homogenization, microwave or ultrasonic action, enzymatic treatments, or alkaline hydrolysis. However, the accessibility of proteins for human consumption depends very much on the thickness of the cell wall, which in some cases, such as *H. pluvialis* in red phase, can be very thick due to the sporopollenin content. Since proteins make up most of the cellular content of *H. pluvialis* microalgae in the green and red phase, *Nannochloropsis* sp. and *S. almeriensis* with percentages up to 32.59% *w*/*w* on a dry basis. Some authors compared different methods of pre-treatment (manual grinding, ultrasonication, alkaline hydrolysis, high-pressure homogenization) for some of these species [101]. They observed that high-pressure homogenization is the best pre-treatment for all tested species obtaining yields between 41% and 78% of proteins and that the composition of non-essential and essential amino acids decreases in protein extracts of the species *H. pluvialis* and *N. oculate* [102].

## 4. Conclusions

The great advantage of microalgae is their ability to produce different compounds such as proteins, carbohydrates, lipids, and carotenoids, with compositions depending on the species. Some microalgae such as *C. vulgaris*, *A. platensis*, *H. pluvialis* and *D. salina* are principally currently marketed in the food, feed, and nutraceutical sectors. On basis on their potentiality to produce in the same time protein (20–40% *w*/*w* on a dry basis), carotenoids (0.30–4% *w*/*w* on a dry basis), lipids (1–15% *w*/*w* on a dry basis) in particular omega-6 and omega-3, they can find greater applications than at present in many markets, such as animal feed and aquaculture, the protein market and cosmetics. *Nannochloropsis* sp. and *S. almeriensis* are promising species for EPA production in omega-3 and lutein production, respectively, in competition with the *Tagetes erecta* plant.

## Figures and Tables

**Table 1:** Moisture and ash contents of *Arthrospira platensis*, *Scenedesmus almeriensis*, * Haematococcus pluvialis* (green phase), *Haematococcus pluvialis* (red phase), *Chlorella vulgaris*, *Dunaliella salina*, *Nannochloropsis* sp.

Composition	*Arthrospira platensis*	*Scenedesmus almeriensis*	*Haematococcus pluvialis* (Green Phase)	*Haematococcus pluvialis* (Red Phase)	*Chlorella vulgaris*	*Dunaliella salina*	*Nannochloropsis* sp.
Moisture *	6.45 ± 0.15	8.89 ± 0.32	5.03 ± 0.12	2.79 ± 0.23	1.92 ± 0.09	6.63 ± 0.25	1.90 ± 0.05
Ash #	5.71 ± 0.32	57.61 ± 2.20	29.49 ± 0.22	4.02 ± 0.22	10.88 ± 0.51	48.74 ± 2.50	8.31 ± 0.42

* % *w*/*w* on wet basis ± SD (*n* = 3); # % *w*/*w* on a dry basis ± SD (*n* = 3).

**Table 2:** Protein, carbohydrates, lipids, total dietary fiber, and carotenoid compositions expressed as expressed as % *w*/*w* on a dry basis ± SD (*n* = 3) of *Arthrospira platensis*, *Scenedesmusalmeriensis*, *Haematococcus pluvialis* (green phase), *Haematococcus pluvialis* (red phase), *Chlorella vulgaris*, *Dunaliella salina*, *Nannochloropsis* sp.

Composition (% *w*/*w* on a Dry Basis ± SD)	*Arthrospira platensis*	*Scenedesmus almeriensis*	*Haematococcus pluvialis* (Green Phase)	*Haematococcus pluvialis* (Red Phase)	*Chlorellavulgaris*	*Dunaliella salina*	*Nannochloropsis* sp.
Protein	46.76 ± 0.95	12.93 ± 0.69	32.59 ± 1.20	25.69 ± 1.27	45.64 ± 1.20	10.03 ± 0.57	26.67 ± 1.10
Carbohydrates	3.32 ± 0.05	4.51 ± 0.41	0.13 ± 0.01	6.30 ± 0.24	5.30 ± 0.50	25.31 ± 1.55	32.05 ± 0.70
Lipids	1.40 ± 0.12	2.05 ± 0.12	3.24 ± 0.11	2.60 ± 0.10	3.13 ± 0.21	3.49 ± 0.10	15.30 ± 0.24
Total dietary Fiber	42.82 ± 1.20	22.60± 1.50	34.56 ± 0.90	58.52 ± 2.56	35.04 ± 1.60	8.97 ± 0.50	17.67 ± 0.80
Carotenoids	<Ldl *	0.30 ± 0.05	<Ldl *	2.87 ± 0.15	<Ldl *	3.46 ± 0.15	<Ldl *

<Ldl * = lower than the detection limit.

**Table 3:** Fatty acids composition (mg/100 g on a dry basis ± SD (*n* = 3)).

Fatty Acid Composition (mg/100 g on a dry basis ± SD)	*Artrhospira platensis*	*Scenedesmus almeriensis*	*Haematococcuspluvialis* (Green Phase)	*Haematococcus pluvialis* (Red Phase)	*Chlorella vulgaris*	*Dunaliella salina*	*Nannochloropsis* sp.
SFAs *							
Tridecanoic acid	nd ^#^	nd ^#^	10.90 ± 0.22	nd ^#^	11.21 ± 0.39	nd ^#^	233.20 ± 0.67
Palmitic acid	253.32 ± 0.73	249.07 ± 0.90	521.73 ± 0.49	506.68 ± 0.27	598.75 ± 2.20	965.00 ± 1.15	26.43 ± 1.50
Pentadecanoic acid	nd ^#^	nd ^#^	nd ^#^	3.22 ± 0.18	nd ^#^	nd ^#^	294.71 ± 0.38
Heptadecanoic acid	nd ^#^	nd ^#^	19.28 ± 0.42	nd ^#^	nd ^#^	nd ^#^	1253.38 ± 2.35
Stearic acid	9.94 ± 0.24	33.83 ± 1.23	130.36 ± 1.98	42.07 ± 0.35	22.14 ± 1.39	567.68 ± 0.56	nd ^#^
Arachidic acid	124.38 ± 0.95	66.22 ± 2.49	nd ^#^	63.12 ± 1.37	nd ^#^	nd ^#^	382.51 ± 1.94
∑ other SFAs	-	-	56.64 ± 1.49	31.64 ± 1.19	29.28 ± 0.94	-	16.71 ± 0.62
∑ SFAs	387.64 ± 1.61	349.11 ± 4.58	738.91 ± 4.40	646.73 ± 2.74	661.37 ± 4.32	1532.68 ± 1.70	2316.54 ± 2.02
MUFAs *							
Palmitoleic acid	38.47 ± 0.54	60.04 ± 0.90	51.23 ± 0.88	4.82 ± 0.61	15.69 ± 0.98	nd #	2588.39 ± 1.79
cis-9-Octadecenoic acid (oleic acid)	65.06 ± 0.96	109.11 ± 1.67	nd ^#^	486.17 ± 0.23	226.43 ± 0.74	567.56 ± 1.29	nd ^#^
Myristoleic acid	16.11 ± 0.94	16.99 ± 1.25	33.65 ± 0.68	nd ^#^	23.19 ± 0.97	nd ^#^	483.93 ± 0.86
Nervonic acid	nd ^#^	nd ^#^	nd ^#^	nd ^#^	nd #	nd ^#^	522.39 ± 0.23
Erucic acid	433.46 ± 0.83	nd ^#^	51.78 ± 0.70	nd ^#^	nd ^#^	nd ^#^	nd ^#^
∑ other MUFAs	-	49.28 ± 1.13	81.54 ± 0.70	52.60 ± 0.62	177.04 ± 1.37	-	44.59 ± 0.85
∑ MUFAs	553.10 ± 1.75	235.42 ± 2.80	218.20 ± 0.93	543.60 ± 1.13	442.35 ± 1.26	567.56 ± 1.29	3639.30 ± 2.77
PUFAs *							
cis-8,11,14-Eicosatrienoic acid	nd ^#^	nd ^#^	nd ^#^	13.94 ± 0.57	nd ^#^	nd ^#^	802.59 ± 0.82
Linoelaidic acid	nd ^#^	nd ^#^	nd ^#^	nd ^#^	nd ^#^	nd ^#^	590.51 ± 1.35
Linoleic acid	76.92 ± 0.51	161.41 ± 0.23	251.61 ± 0.50	714.43 ± 1.06	691.87 ± 0.77	519.75 ± 0.63	nd ^#^
γ-Linolenic acid	38.40 ± 0.77	370.84 ± 0.84	723.07 ± 0.21	205.85 ± 0.45	574.54 ± 0.35	536.22 ± 0.12	nd ^#^
Arachidonic acid	nd ^#^	nd ^#^	nd ^#^	173.34 ± 0.27	nd ^#^	nd ^#^	nd ^#^
cis-5,8,11,14,17-Eicosapentaenoic acid	nd ^#^	nd ^#^	nd ^#^	nd ^#^	nd ^#^	nd ^#^	3650.82 ± 1.97
∑ others PUFAs	-	-	-	-	-	-	-
∑ PUFAs	115.31 ± 0.98	532.25 ± 1.00	974.68 ± 0.59	1107.55 ± 1.75	1266.40 ± 0.47	1055.97 ± 0.75	5043.91 ± 3.28

* SFAs: saturated fatty acids; MUFAs: monounsaturated fatty acids; PUFAs: polyunsaturated fatty acids; ^#^ nd = not detected.

**Table 4:** Microalgae composition (% *w*/*w* on dry ± SD *): comparison with literature.

Microalgae	Protein	Carbohydrates	Lipids	Total Dietary Fiber	Reference
*Arthrospira platensis*	50–63	7.7–22.2	-	-	[4,63,64,65]
	42.08 ± 0.10	21.5	-	8.5	[5]
	-	-	4–9	-	[66]
	46.76 ± 0.95	3.32 ± 0.05	1.40 ± 0.12	42.82 ± 1.20	This Study
*Scenedesmus almeriensis*	49.4–55	-	-	-	[49,59]
	-	24.6	-	-	[9]
	-	-	1.58	-	[66]
	−12.93 ± 0.69	4.51 ± 0.41	2.05 ± 0.12	22.60 ± 1.50	This Study
*Haematococcus pluvialis (green phase)*	32.59 ± 1.20	0.13 ± 0.01	3.24 ± 0.11	34.56 ± 0.90	This Study
*Haematococcus pluvialis (red phase)*	10.2–17	-	-	-	[7,51]
	25.69 ± 1.27	6.30 ± 0.24	2.60 ± 0.10	58.52 ± 2.56	This Study
*Chlorella vulgaris*	20–60.38	-	-	-	[67]
	25.50–48.19	59.71	-	16.37–25.95	[68,69]
	-	-	12–26	-	[66]
	45.64 ± 1.20	5.30 ± 0.50	3.13 ± 0.21	35.04 ± 1.60	This Study
*Dunaliella salina*	55	25–40	-	-	[49]
	-		17	-	[70]
	10.03 ± 0.57	25.31 ± 1.55	3.49 ± 0.10	8.97 ± 0.50	This Study
*Nannochloropsis* sp.	41.6–42.1	16.7–18.6	-	-	[5]
	28.8 ± 0.63	28.7 ± 0.48–40.4	-	-	[71]
	-	0.39	-	-	[49]
	-	-	>40	-	[72]
	-	-	25.6–30	-	[72]
	26.67 ± 1.10	32.05 ± 0.70	-	17.67 ± 0.80	This Study

* SD = standard deviation.

**Table 5:** Cereals composition (% *w*/*w* on dry weight).

Cereals	Moisture	Ash	Proteins	Carbohydrates	Lipids	Total Dietary Fiber	Carotene
Wheat	14	1.4	10.6	61.6–69.7	1.4	10.5–14.4	0.02
Barley	14	1.9	11	55.8	3.4	4.7	n.a. *
Rye	14	1.8	8.7	60.9–71.8	1.5	13.1	0
Brown Rice	14	1.4	7.3	64.3–71.1	2.2	4	0
Sorghum	14	2.6	8.3	57.4–62.9	3.9	13.8	10
Oats	14	2.3	9.3	62.9–63	5.9	5.5	0
Maize	14	1.4	9.8	60.9–63.6	4.9	9	0.37

* n.a. = not available.

**Table 6:** Main microalgae biomass and products applied in food, feed, and food supplements, authorized by EU regulations and the FDA [12,78].

Products Name	Application	Characteristics	Regulations	Price
*A. platensis* extract	Additive in food and drugs, coloring agent, dietary supplements	Filtered aqueous extract from dried biomass, principal colorant phycocyanin	CFR-TITLE 21-FDA	US$29.75/120 mL
				€70–200 kg [81]
Dried algae meal (genus *Spongiococcum*)	Additive for chicken skins, and eggs	A mixture of dried biomass, molasses, corn step liquor after fermentation	CFR-TITLE 21-FDA	US$50/kg [82]
Dried *T. chuii*	Novel food and food supplement; ingredient for sauce, special salt and condiment	Humidity ≤7.0%	Regulation (EC) 2017/2470 Directive 2002/46/CE	US$50/kg [82]
		Protein 35–40%		
		Carbohydrates 30–32%, ash 14–16%		
		Fiber 2–3%		
		Fat 5–8%		
		SFAs 29–31%		
		MUFAs 21–24%		
		PUFAs 44–49%		
		Iodine ≤15 mg/kg		
*O. aurita*	Novel food, food additive in flavored pasta, fish soups, marines terrines, broth preparation, crackers, frozen breaded fish	Diatom with silicon 3.3%	Regulation (EC) 2017/2470	
*H. pluvialis* meal	Color additive for fish feed (salmonids)	Dry and comminuted solid biomass containing not less than 1.5% astaxanthin	CFR-TITLE 21-FDA	US$489/kg [83]
*C. vulgaris*	Food source falling into the generally recognized as safe (GRAF) category		FDA (2016) Summary: Substances generally regarded as safe (Final Rule). U.S. Food and Drug Administration (HHS).	50–600/kg [84]
Protein powder and lipid ingredient derived from *C. vulgaris*	Food source falling into the generally recognized as safe (GRAF) category		FDA (2016) Summary: Substances generally regarded as safe (Final Rule). U.S. Food and Drug Administration (HHS).	€8.30/kg (Production cost) [85]
Beta-carotene (E 160 IV) (73.95 or 73.1095 beta-carotene)	Additive for food, feed, for all drugs including those for eyes, dietary supplements and cosmetics	E-160 IV: oil essential extract from *D. salina*; beta-carotene content not less than 20% and other carotenes may be present; 73.95: Physical state, solid, 1% percent solution in chloroform; Beta-carotene content 96–100%	Regulation (EC) 231/2012	US$50–500/ kg
			Regulation (EC) 1103/2015	
			Regulation (EC) 1170/2009	€150/kg [86]
			CFR-TITLE 21-FDA	
Astaxanthin	Color additive for fish feed	Physical state, solid, 0.05 percent solution in chloroform Astaxanthin content minimum 96%	CFR-TITLE 21-FDA	€99/kg (astaxanthin content: 0.5%) [83]
Astaxanthin-rich oleoresin from *H. pluvialis*	Novel food and food supplement	Astaxanthin is extracted by CO_2_-SFE or diluted ethyl acetate using olive oil, sunflower oil or medium chain triglycerides.	Regulation (EC) 2017/2470 Directive 2002/46/CE	€499/kg (astaxanthin content: 5%) [83]
		Protein 0.3–4.4%		
		Carbohydrates 0–52.8%		
		Ash 0.0–4.2%		
		Fiber <1%		
		Fat 42.2–99%		
		Total astaxanthin 2.9–11.1%		
		All-trans astaxanthin 79.9–91.5%		
		9-cis astaxanthin 0.3–17.3%		
		13-cis astaxanthin 0.2–7.0%		
		Beta-carotene 0.01–0.3%		
		Lutein 0–1.8%		
		Canthaxanthin 0–1.30%		
Algal oil from *Ulkenia* sp.	Novel food for bakery products, cereal bars, non-alcoholic beverages	Non-saponificable fraction ≤ 4.5%	Regulation (EC) 2017/2470	US$80–160/kg [86]
		Trans fatty acids ≤ 1.0%		
		DHA content ≥ 32%		
*Schizochytrium* sp. Oil rich in DHA and EPA	Novel food and food supplement for adult, pregnant and lactating women, baby food	Non-saponificable fraction ≤ 3.5%	Regulation (EC) 2017/2470 Directive 2002/46/CE	US$80–160/kg [86]
		Trans fatty acids ≤ 1.0%		
		DHA content ≥ 22.5%		
		EPA content ≥ 10%		
*Nannochloropsis* sp.derived oil (rich in EPA)	Safe for use in dietary supplements		FDA (2015) US Food and Drug Administration New Dietary Ingredient Notification Report #826	US$80–160/kg [86]
